# Association of HPV genotypes with external anogenital warts: a cross sectional study

**DOI:** 10.1186/s12879-019-4005-4

**Published:** 2019-05-02

**Authors:** Rana Al-Awadhi, Nawaf Al-Mutairi, Ahmed N. Albatineh, Wassim Chehadeh

**Affiliations:** 1 Department of Medical Laboratory Sciences, Faculty of Allied Health Sciences, Kuwait University, P.O. Box 31470, 90805 Sulaibikhat, Kuwait; 2 Dermatology Unit, Department of Medicine, Faculty of Medicine, Kuwait University, Kuwait City, Kuwait; 3 Department of Community Medicine and Behavioral Sciences, Faculty of Medicine, Kuwait University, Kuwait City, Kuwait; 4 Virology Unit, Department of Microbiology, Faculty of Medicine, Kuwait University, Kuwait City, Kuwait

**Keywords:** Anogenital warts, HPV, Prevalence, Multiple regression

## Abstract

**Background:**

This study was undertaken to determine the distribution of type-specific human papillomavirus (HPV) in external anogenital warts, and the correlation with clinical presentation of warts and demographic data of patients.

**Methods:**

Genital warts specimens were obtained from 129 men and 27 women attending a dermatology clinic, who had been advised surgical excision. The tissues were fixed and screened for HPV DNA by using real-time PCR. HPV genotype was determined by PCR-based sequencing.

**Results:**

Sixteen different HPV genotypes were detected, comprising 4 oncogenic HPV genotypes (16, 18, 33, 38), 2 low-risk HPV types (LR) (6, 81), HPV 9, and other types associated with common warts (1a, 2, 4, 7, 27b, 27, 57b, 57c, 65). Oncogenic HPV types were found in 34.62% of patients, LR HPV types in 14.4%, HPV 9 in 0.64%, and common warts type in 50.6%. The prevalence of HPV infection with a single type was 88.4, 9.0% for two types, and 2.6% for three types. Multiple logistic regression model showed that age, gender, nationality, number of warts, size of each wart, and positive history of wart in sexual partner, were not predictors of HPV type. However, patients with anogenital warts of one to six months duration were three times more likely to have oncogenic HPV infection compared to those with less than one month.

**Conclusions:**

This study shows that oncogenic HPV types are detected in around 35% of patients with genital warts, and are prevalent in warts of one to six months duration.

## Background

Anogenital condyloma acuminata are warts around the genital and anal areas. More than 90% of anogenital warts are caused by HPV type 6 and 11 [13, 28]. In women, external anogenital warts are always accompanied by flat or exophytic warts of the vagina and cervix [26]. In men, genital HPV infection is manifested by the presence of condyloma acuminata of anogenital areas, penile intraepithelial neoplasia and penile carcinoma [23]. Since HPV is a sexually transmitted virus, the incidence of external anogenital warts is also associated with the sexual behavior of patients [14, 26].

Limited reports are available on HPV infection and anogenital warts locally and worldwide. In the Kuwaiti context’, one report published in 2004 by Al-Fouzan and Al-Mutairi [3] presented an overview of the incidence of sexually transmitted infections (STI) in Kuwait. They found that the incidence of genital warts (condyloma accuminata) has shown steady increase from 2.04% of STI cases in 1998 to 2.3% in 2002. The results were based on clinical diagnosis. Another report was carried out in 2007 by Al-Mutairi et al. [4] to determine the clinical patterns, sociodemographic factors, and sexual practices of patients with STI who attended a government hospital in Kuwait found that 13.7% of 1096 patients included in the study had genital warts.

We have previously conducted a study to determine the prevalence and type specific distribution of HPV in women with normal cervical cytology samples in Kuwait. HPV DNA was detected in 2.4% of women with cytologically normal cervices; low-risk (LR) HPV types were detected in 71.8% of the infected samples, high-risk (HR) HPV types in 32.3%, and intermediate-risk (IR) types in 7% [2]. We have also reported the presence of HPV DNA in 51% of abnormal cervical cytology samples; HPV16 had the highest prevalence (24.3%), followed by HPV11 (13.8%), HPV66 (11.2%) and HPV33 (9.9%), [1].

Three different prophylactic HPV vaccines are currently approved. Gardasil/Silgard which targets HPV6, 11, 16, and 18; Cervarix which targets HPV 16 and 18; and Gardasil 9 which targets HPV6, 11, 16, 18, 31, 33, 45, 52, and 58, reviewed in Gupta et al., 2018 [17].

In order to investigate whether the HPV types associated with genital warts are covered by the current licensed HPV vaccines, the HPV types detected in the genital warts were identified by direct sequencing. Demographic data and warts characteristics were used in a regression model to predict the HPV type.

## Methods

### Study design and participants

A cross sectional study that uses a non-probabilistic sample was conducted. Patients attending dermatology clinics at Farwania Health District Area were recruited in the study consecutively from November 2016 to May 2017. Immunocompetent patients with previous clinical diagnosis of external anogenital warts scheduled for cryotherapy or laser treatment were asked to sign the consent form after a verbal explanation from the dermatologist soliciting their participation. At the time of sampling, wart specimens were collected in universal transporting medium (HealthLink Inc., Copan Italia S.p.A., Italy), and stored at − 80 °C. Ethical approval was obtained from the Health Sciences Center Ethical Committee (Number: VDR/EC/2310) and Ministry of Health.

Demographic and clinical information were taken from each patient, including: age (years), gender (male, female), nationality (Kuwaiti, non-Kuwaiti), number of warts (one, two to four, and at least five), duration of the presence of warts (< one month, one to six months, seven to 12 months, and more than 12 months), size of each wart (in centimeter) (< one, one, between one and two, more than two), partners has warts (yes, no), and if warts were subject to prior treatment (yes, no). None of the participants received HPV vaccination, because HPV vaccination has not yet been implemented in Kuwait, although its implementation is currently being discussed at the Ministry of Health.

### DNA extraction and controls

Received tissue samples were stored at − 80 °C. Due to the large number of samples received, all warts per patient were minced together and were subjected to one DNA isolation. Tissue was minced with sterile scissor and forceps on a Petri dish and about 25-mg of tissue was weighed. The tissue was washed in phosphate buffered saline (PBS) and transferred into 1.5-ml Eppendorf tubes. Genomic DNA was extracted from tissue samples using a QIAmp DNA Mini kit (Qiagen, USA) according to the manufacturer’s instructions.

HPV type 2, HPV type 1 and HPV type 16 vectors (American Type Culture Collection, Manassas, VA, USA) were used as controls in PCR experiments.

### Real time PCR

The real-time PCR assay was carried out to screen for HPV DNA in the genital warts. Five microlitres (5-μl) of the extracted DNA was combined with 12.5-μl of 2X Syber Green master mix (Applied Biosystems, Foster City, CA, USA) containing ROX as a passive reference, and 10 pmol of forward and reverse primers (10-uM, described below). All sets of the primers were custom synthesised by Thermo Fisher Scientific, Waltham, Massachusetts, USA. The mixture was made up to 25-μl volume with nuclease free water (Ambion, Austin, TX, USA). In order to reduce the number of false positive or negative results, samples were analyzed in duplicate on a 96 optical well reaction plate (Applied Biosystems, Foster City, CA, USA). Positive and negative controls were included in each amplification batch. In order to quantify the HPV in the samples, a five to 10 fold serial dilution of the known positive control DNA was performed alongside the samples. Real-time PCR amplification was carried out in ABI 7500 real-time PCR (Applied Biosystems, Foster City, CA, USA).

The real-time PCR assay was carried out on three different plates. The first plate was used to determine the integrity of the target DNA by β-globin PCR assay, amplifying a target of 268- bp fragment, as described previously by Lum and Le Marchand in 1998 [20]. The nucleotide sequence of β-globin primers is as follows: β-globin forward PCR primer: 5′–TGGGTTTCTGATAGGCACTGACT-3′. β-globin reverse PCR primer: 5′-AACAGCATCAGGAGTGGACAGAT-3′.The PCR amplification was initiated at 95 °C for ten minutes and completed by 45 amplification cycles (denaturation at 95 °C for 15 s, annealing at 55 °C for 45 s and extension at 65 °C for one minute).

The second plate was used to screen for the presence of HPV infection using MY11/GP6 primers from the HPV L1 ORF [6]. The nucleotide sequences of MY11/GP6 primers [6] are as follows: MY11 forward primer: 5′- GCM CAG GGW CAC AAY AAT GG -3′ and GP6 reverse primer: 5′- GAAAAATAAACTGTAAATCATATTC-3′. The expected size of amplified fragment is 185-bp. The PCR amplification was initiated at 95 °C for ten minutes and completed by 45 amplification cycles (denaturation at 95 °C for 15 s, annealing at 45 °C for 45 s and extension at 65 °C for one minute).

The third plate was used to screen for the presence of HPV infection using HVP2/B5 primers from the HPV L1 ORF [18]. The nucleotide sequences of HVP2/B5 primers are as follows: HVP2 forward primer: 5′- TCN MGN GGN CAN CCN YTN GG -3′; B5 reverse primer: 5′- AYN CCR TTR TTR TGN CCY TG -3′. The expected size of the amplified fragment is 650-bp. The PCR amplification was initiated at 95 °C for ten minutes and completed by 45 amplification cycles (denaturation at 95 °C for 15 s, annealing at 50 °C for 45 s and extension at 65 °C for one minute).

Fluorescence spectra were recorded during the elongation phase of each PCR cycle. Sequence Detection Software (SDS v1.7) of ABI 7500 real-time PCR was used to generate the amplification curve for each reaction. A dissociation curve was generated after each reaction to differentiate between specific and non-specific amplicons. On the basis of the amplification curve, all samples with HPV amplification starting at any cycle and up to cycle number 40 (with a cut-off line of 0.2) were selected for the analysis. Only samples with a dissociation curve between 70 °C and 80 °C and with a derivative value between 0.100–0.500 were considered HPV DNA positive.

### Sanger sequencing

Samples showing positive amplification for the presence of HPV by MY11/GP6 were subjected to conventional PCR assay. The expected HPV genotype spectrum to be detected from mucosal warts using MY11/GP6 includes, HR HPV genotypes: 9, 16, 18, 26, 31, 33, 35, 38, 39, 45, 51, 52, 55, 56, 58, 59 and 68, LR HPV genotypes: 6, 11, 40, 42, 53, 54, 57, 66 and 81 [16].

HPV DNA in warts was amplified by nested PCR prior to sequencing, using the AmpliTaq Gold Master Mix (Applied Biosystems, Foster City, CA, USA) HVP2/B5 primers were used in the first PCR, and CN1F/CN1R, CN2F/CN2R, CN3F/CN3R and C4F/C4R primers [18] in the second PCR. The first PCR amplification was initiated at 95 °C for ten minutes and completed by 35 amplification cycles (denaturation at 95 °C for 15 s, annealing at 50 °C for 45 s and extension at 68 °C for one minute). The second PCR amplification was carried out using 3 μl of the first PCR product, and the same cycling conditions. The expected broad-spectrum HPV genotypes to be detected from cutaneous warts using HVP2/B5 and CN1F/CN1R, CN2F/CN2R, CN3F/CN3R and C4F/C4R primers includes HPV types from genera alpha (HPV 2, 3, 7, 10, 27, 28, 29, 40, 43, 57, 77, 91 and 94), gamma (HPV 4, 65, 95, 48, 50, 60 and 88), mu (HPV 1 and 63), and nu (HPV 41) [12].

The PCR products were purified using a PCR purification kit (NucleoSpin Extract II PCR Purification Kit, Macherey-Nagel GmbH & Co.KG, Düren, Germany) as per the manufacturer’s instructions. They were then subjected to Sanger sequencing reaction using BigDye terminator v3.1 cycle sequencing mix (Applied Biosystems, Foster City, CA, USA), and the nested PCR primers described above. Post sequencing PCR purifications were performed to remove unbound fluorescent dye deoxy terminators using BigDye XTerminator™ Purification kit (Applied Biosystems, Foster City, CA, USA). The samples were denatured for 2 min at 95 °C and immediately chilled on ice and loaded on an ABI 3100 Genetic Analyzer (Applied Biosystems, Foster City, CA, USA). Samples were electrophoresed on a 50-cm capillary array using POP 6 polymer (Applied Biosystems, Foster City, CA, USA) as a separation medium.

The samples were analyzed using Sequencing Analysis software v 3.7 (Applied Biosystems, Foster City, CA, USA). The HPV sequence alignment was performed with sequences presented in the GenBank database using BLASTn software (https://blast.ncbi.nlm.nih.gov/) and the Los Alamos Data National Laboratory Theoretical Biology and Biophysics HPV database (https://pave.niaid.nih.gov/).

When obtained sequences were of poor quality due to low yield of PCR product, the PCR products were cloned into the pGEM-T Easy Vector (Promega, Madison, WI) following purification with the Wizard SV Gel and PCR Clean-Up System kit (Promega), and the sequencing of inserts was performed using M13 forward and reverse primers.

### Statistical analysis

Data of the clinical diagnosis, demographic data and virological analysis were tabulated and analyzed using SPSS statistical software ‘Statistical Package for Social Sciences, SPSS version 25.0’ (IBM Corp, Armonk, NY, USA). Descriptive statistics: frequencies/percentages, measures of center and dispersion were calculated. For testing equality of means, the two-sample t-test was used for all continuous variables when normality was held, otherwise Mann Whitney U test was used. For comparing means of three groups, either analysis of variance or Kruskal-Wallis test was used depending on the assumptions being satisfied. Pearson chi square test for independence or Fisher exact test were used to test associations between two categorical variables when the assumptions were satisfied. To model a binary outcome with a set of covariates, logistic regression was implemented, and crude and adjusted odds ratios and their 95% confidence intervals were estimated. All tests were two tailed and a *P* value less than 5% was considered statistically significant.

## Results

External anogenital wart samples obtained from 156 patients attending outpatient dermatology clinics were included in the study. Anogenital warts included warts taken from penis, pubic area, anus, perianal area and vulva. The mean of their ages was 35.36 years, Standard Deviation (SD) was 9.59, median was 33 years, and range was 16–56 years. The mean of male patient ages was 34 and the mean of female ages was 42.

Data revealed that the majority of patients (98.1%) had more than one wart for a duration of more than one month, and of size between one and two centimeters. Of note, 75.6% of the patient partners had no anogenital warts (Table 1).

**Table 1:** Clinical and demographic characteristics of patients with anogenital warts (*N* = 156)

Characteristic	*n* (%)
Gender	
Male	129 (82.7)
Female	27 (17.3)
Nationality	
Kuwaiti	79 (50.6)
Non-Kuwaiti	77 (49.4)
Number of Warts	
One	3 (1.9)
Two – Four	89 (57.1)
At least Five	64 (41.0)
Duration of presence of each Wart	
Less than one month	33 (21.2)
One – six months	80 (51.3)
Seven – Twelve months	29 (18.6)
More than 12 months	14 (9.0)
Size of each Wart	
Less than one Cm^1^	36 (23.1)
One Cm	37 (23.7)
One – two Cm	70 (44.9)
More than two Cm	13 (8.3)
Wart subject to prior treatment	
No	91 (58.3)
Yes	65 (41.7)
Partner has Warts	
No	118 (75.6)
Yes	38 (24.4)

Data are numbers of patients (%). ^1:^ Centimeter

The analysis of the sequencing results showed that there were 16 different HPV genotypes detected in this study, including HPV genotype 1a, 2, 4, 6, 7, 9, 16, 18, 27, 27b, 33, 38, 57b, 57c, 65 and 81. Table 2 presents the prevalence of HPV genotypes according to whether the patient was infected with single, double, or triple HPV types. The prevalence of oncogenic HPV genotypes (HPV 16, 18, 33 and 38) was 54/156 (34.62%), whereas that of LR HPV genotype (HPV 6 and 81) was 22/156 (14.1%) and common warts viruses (1a, 2, 7, 27b, 27, 57b, 57c, 65) was 50.6%. HPV infection with a single type, two types, and three types was found in 88.4, 9, and 2.6% of patients, respectively.

**Table 2:** HPV type-specific prevalence in patients with anogenital warts (*N* = 156)

Infection with a single HPV type *n* (%)		Infection with two HPV types *n*(%)		Infection with three HPV types *n* (%)	
16	44 (28.2)	1a + 57c	2 (1.3)	1a + 2 + 4	1 (0.64)
27b	23 (14.7)	6 + 57c	2 (1.3)	1a + 2 + 16	1 (0.64)
57c	22 (14.1)	16 + 57c	2 (1.3)	1a + 57c + 65	1 (0.64)
6	16 (10.3)	1a + 27b	1 (0.64)	9 + 27b + 65	1 (0.64)
2	15 (9.6)	1a + 33	1 (0.64)		
65	7 (4.5)	1a + 16	1 (0.64)		
1a	2 (1.3)	2 + 27b	1 (0.64)		
18	2 (1.3)	6 + 2	1 (0.64)		
57b	2 (1.3)	6 + 27b	1 (0.64)		
7	1 (0.64)	6 + 65	1 (0.64)		
9	1 (0.64)	16+ 27b	1 (0.64)		
27	1 (0.64)	16 + 2	1 (0.64)		
38	1 (0.64)				
81	1 (0.64)				

Data are numbers of patients (%). Listed are the most prevalent HPV types, other types occurred in multiple infection including HPV 4 and HPV 33

The association between oncogenic HPV genotypes (HPV 16, 18, 33 and 38) with demographic data and warts characteristics is shown in Table 3. The mean age of patients with oncogenic viruses was 36 years, SD of 9.8, median, 33 years and range, 18–56 years. The mean age of patients with non-oncogenic viruses was 35 years, SD of 7.9, median, 33 years, and range, 16–56 years, and the mean difference in age was statistically significant (PV = 0.006). The multiple logistic regression model (Table 4) indicated that age, gender, nationality, number of warts, size of each wart and if partner has warts were not significantly associated with oncogenic HPV infection. However, the adjusted Odds Ratio (OR) for detecting oncogenic HPV type in a wart of one to six months duration, was three times higher than that of a wart with less than one month duration.

**Table 3:** Association of demographic and warts characteristics with oncogenic HPV detection (*N* = 156)

Characteristic	Oncogenic HPV *n *(%)	Non-Oncogenic HPV *n *(%)	*P*. Value
Gender			0.462^3^
Male	43 (33.3)	86 (66.7)	
Female	11 (40.7)	16 (59.3)	
Nationality			0.219^3^
Kuwaiti	31 (39.2)	48 (60.8)	
Non-Kuwaiti	23 (29.9)	54 (70.1)	
Number of Warts			0.614^2^
One	1 (33.3)	2 (66.7)	
Two – Four	34 (38.2)	55 (61.8)	
At least Five	19 (29.7)	45 (70.3)	
Duration of presence of each Wart			0.162^3^
Less than one month	6 (18.2)	27 (81.8)	
One – six months	31 (38.8)	49 (61.3)	
Seven – Twelve months	12 (41.4)	17 (58.6)	
More than 12 months	5 (35.7)	9 (64.3)	
Size of each Wart			0.047^4^
Less than one Cm^1^	9 (25.0)	27 (75.0)	
One Cm	9 (24.3)	28 (75.7)	
One – two Cm	31 (44.3)	39 (55.7)	
More than two Cm	5 (38.5)	8 (61.5)	
Wart subject to prior treatment			0.060^3^
No	26 (28.6)	65 (71.4)	
Yes	28 (43.1)	37 (56.9)	
Partner has Warts			0.469^3^
No	39 (33.1)	79 (66.9)	
Yes	15 (39.5)	23 (60.5)	

Data are numbers of patients (%).^1:^ Centimeter. Oncogenic HPV if it is positive in one of HPV types: 16, 18, 33 and 38. Non-oncogenic HPV if it is positive in one of HPV types: 1a, 2, 6, 7, 9, 27, 27b, 57b, 57c, 65 and 81. ^2^Fisher exact test, ^3^Pearson Chi square test, ^4^ Chi square test for trend

**Table 4:** ^a^Adjusted association of demographic and warts characteristics with oncogenic HPV detection (*N* = 156)

Covariate	Crude OR (95% CI)	*P*-Value	Adjusted OR (95% CI)	*P*- Value
Age (years)	1.01 (0.98, 1.05)	0.467	1.02 (0.98, 1.06)	0.447
Gender				
Female	1 (ref)		1 (ref)	
Male	0.73 (0.31, 1.70)	0.463	0.76 (0.24, 2.38)	0.639
Nationality				
Non-Kuwaiti	1 (ref)		1 (ref)	
Kuwaiti	1.52 (0.78, 2.95)	0.220	1.01 (0.42, 2.45)	0.984
Number of Warts		0.552		0.221
One	1 (ref)		1 (ref)	
Two-Four	1.24 (0.11, 14.16)	0.865	2.32 (0.15, 34.95)	0.544
At least five	0.84 (0.07, 9.88)	0.893	1.13 (0.07,17.47)	0.929
Duration of Wart (months)		0.184		0.114
Less than one	1 (ref)		1 (ref)	
One – Six	2.85 (1.06, 7.68)	0.039	3.06 (1.03, 9.09)	0.045
Seven – Twelve	3.18 (1.01, 10.06)	0.049	1.12 (0.25, 5.05)	0.879
More than Twelve	2.50 (0.61, 10.20)	0.202	1.70 (0.32, 9.09)	0.535
Size of each Wart (Cm)^b^		0.109		0.258
< 1	1 (ref)		1 (ref)	
1	0.964 (0.333, 2.80)	0.947	0.61 (0.19, 2.03)	0.424
Between 1 and 2	2.39 (0.98, 5.81)	0.056	1.66 (0.57, 4.86)	0.353
> 2	1.88 (0.49, 7.22)	0.361	1.43 (0.31, 6.74)	0.649
Wart subject to prior treatment				
No	1 (ref)		1 (ref)	
Yes	1.89 (0.97, 3.69)	0.062	1.63 (0.72, 3.70)	0.246
Partner has Warts				
No	1 (ref)		1 (ref)	
Yes	1.32 (0.62, 2.81)	0.470	1.31 (0.53, 3.22)	0.563

^a^The binary outcome is the results of HPV sequencing being oncogenic genotype (1 = Yes, 0 = No), Oncogenic HPV genotypes include: 16, 18, 33 and 38

^b^Centimeter

## Discussion

This study aimed to investigate the relationship between the type specific distribution of HPV genotypes and clinical characteristics of patients with HPV-infected anogenital warts. It is among few that correlate clinical presentation of warts and demographic data of patients with HPV infection. Unexpectedly, for single type infection, HPV 16 was the most common HPV genotype detected in anogenital warts (28.2%), while HPV 6 prevalence was only 10.3%. As commonly known, LR HPV 6 and HPV 11 are mostly encountered for anogenital warts and HR HPV 16 is always encountered in lower prevalence [5, 7–9].

The authors were surprised to find that HPV 11 was not detected in this cohort. This could be due to the low prevalence of this HPV genotype in anogenital warts. This study is the first of its kind in the Arabian Gulf region further studies are needed in Kuwait and in the region to confirm the outcome of this study. Around 83% of patients in this study were men, due to the fact that, in Kuwait, most women with external genital warts are referred to a gyneacology clinic. The findings of this study show that the mean age of patients infected with HPV (35.3 years) or with HR HPV genotypes (36.2 years) was higher than what is documented worldwide [15].

HPV vaccines were first developed to reduce the number of HPV related cancers and precancerous changes in women. For long-term benefit, it was shown that HPV vaccination reduced the number of HPV related diseases in women, including condyloma acuminata of the vagina, vulva and, of course, perianal areas and cervical intraepithelial neoplasia; and, in men, including anogenital condyloma acuminata and penile intraepithelial neoplasia [10, 24]. Gardasil nonavalent HPV vaccine was designed to target HPV genotypes 6, 11, 16, 18, 31, 33, 45, 52 and 58. According to the outcome of this study, about 50% of anogenital warts are infected with HPV genotypes 6, 16, 18 and 33, and the use of nonavalent HPV vaccine for both sexes would result in the reduction of about 50% of anogenital warts in the studied population which will help in reducing the morbidity of having anogenital warts, and the cost of treatment.

This study shows that the risk of having a genital wart infected with HPV types 16, 18, 33 or 38 increases in a wart of one to six months duration. This could be due to the fact that HR HPV genotypes, especially HPV 16, have longer time to clearance than other genotypes [21]. Other studies linked the development of genital warts and HPV infection to factors related to patients’ sexual behavior, including number of partners [22], men having sex with men [27] and having sex at a young age [11]. Other studies showed that patients who smoke cigarette [25] and marijuana [19] were at high risk of having HPV infection.

## Conclusions

This study shows that oncogenic HPV types are detected in around 35% of patients with genital warts, and are prevalent in warts of one to six months duration.

## Abbreviations

bp: Base pair
Cm: Centimeter
HPV: Human papillomavirus
HR: High-risk
IR: Intermediate-risk
LR: Low-risk
OR: Odds Ratio
ORF: Open reading frame
PBS: Phosphate buffered saline
SD: Standard Deviation
STI: Sexually transmitted infections
μl: Microliters
